# 

**Published:** 1891-01

**Authors:** 


### CONTENTS.

| EDITORIALS. | PAGE |
|---|---|
| Salutatory, | 1 |
| " | 4 |
| Prof. Koch's Discovery, | 5 |
| Dr. Swan's Theory, | 5 |
| The Development of Nosode Practice in the Old School, | 6 |
| The Future of Medicine, | 8 |
| **MISCELLANEOUS.** | |
| How to Use a Repertory. E. J. L, | 9 |
| Action and Reaction. Proceedings of I. H. A., | 14 |
| Nuggets. C. Carleton Smith, M. D., | 16 |
| British Medicinal Plants. Alfred Heath, M. D., | 19 |
| An Instructive Lesson. J. H. Jackson, M. D., | 21 |
| Hiccough. Wm. Steinrauf, M. D., | 24 |
| Syphilinum. Chas. B. Gilbert, M. D., | 24 |
| Gonorrhœa and Homœopathy—A Defence of Dr. T. F. Allen. J. C. White, M. D., | 25 |
| Gonorrhœa and Homœopathy. Robt. Farley, M. D., | 30 |
| Gonorrhœa and Homœopathy. S. A. Kimball, M. D., | 31 |
| Dr. Preston's Case of Syphilis. Mahlon Preston, M. D., | 36 |
| Grafts. Frank S. Davis, M. D., | 38 |
| The Teaching of Homœopathy in the Colleges. Edward F. Brady, M. D., | 39 |
| Homœopathy Going to the Bow-wows. *Eclectic Medical Journal,* | 40 |
| **BOOK NOTICES.** | |
| A Treatise on Headache and Neuralgia. By J. Leonard Corning, M. D., | 42 |
| State Board of Health Bulletin of Tennessee, | 42 |
| Report of Committee on Vital Statistics, | 43 |
| The Disposal of Sewage of Public Edifices, | 43 |
| The Dangers Arising from Public Funerals, | 43 |
| Transactions of California State Homœopathic Society, | 43 |
| Homœopathy and Blood-Letting. By W. B. Clarke, M. D., | 43 |
| The Medical Argus, | 44 |
| Census Bulletins 13, 14, and 15, | 44 |
| Report of Bureau of Organization, etc. American Institute of Homœ- | 44 |
| opathy, | 44 |
| The Medical Bulletin Visiting List, | 44 |
| The Physician's All-Requisite Time and Labor-Saving Account Book, | 45 |
| Post-Mortems. By A. H. Newth, | 45 |
| **NOTES AND NOTICES.** | |
| Women's Homœopathic Hospital—Education in Homœopathy—The Open Court Publishing Company, | 46 |

